# High Molecular Weight Silk Fibroin Prepared by Papain Degumming

**DOI:** 10.3390/polym12092105

**Published:** 2020-09-16

**Authors:** Yanfei Feng, Jiaming Lin, Longxing Niu, Ying Wang, Zhiling Cheng, Xiaoxiao Sun, Mingzhong Li

**Affiliations:** National Engineering Laboratory for Modern Silk, College of Textile and Clothing Engineering, Soochow University, Suzhou 215123, China; fengyanfei0123@126.com (Y.F.); jiaminglin1@126.com (J.L.); niu18860902006@163.com (L.N.); wangying9518@163.com (Y.W.); C1473537105@163.com (Z.C.); 20185215041@stu.suda.edu.cn (X.S.)

**Keywords:** silk, papain, degumming, silk fibroin, molecular weight

## Abstract

A major challenge for the silk textile industry and for the process of silk-based biomaterials is to find a degumming method that can completely remove sericin while avoiding obvious hydrolysis damage to the silk fibroin. In this study, papain was used to degum *Bombyx mori* silk fibers under nearly neutral conditions based on the specificity of papain to sericin. The degumming efficiency was investigated, as well as the mechanical properties and molecular weight of the sericin-free silk fibroin. The results indicated that increasing the papain concentration aided in sericin removal, as the concentration increased to 3.0 g/L, the degummed fibers showed a clean, smooth surface morphology and exhibited a yellow color when stained by picric acid and carmine, confirming the complete removal of sericin from silk fibroin. Furthermore, an analysis of the amino acid composition indicated that the silk fibroin suffered less damage because papain specifically cleaved the binding sites between L-arginine or L-lysine residue and another amino acid residue in sericin, leading to a significantly higher molecular weight and improved tensile strength compared to traditional sodium carbonate degumming. This study provides a novel degumming method which cannot only completely remove sericin, but also maintain the original strong mechanical properties and high molecular weight of silk fibroin.

## 1. Introduction

*Bombyx mori* silk, as a natural protein fiber, has been extensively used in the textile industry for thousands of years, which is greatly appreciated for its desirable physical characteristics, such as good mechanical properties, a pearly luster, a soft texture, and an elegant draping effect [1,2]. In recent years, increasing attention has been focused on the application of silk fibroin (SF) in numerous biomedical applications involved in controlled drug delivery, tissue engineering scaffolds, and implantable devices due to a combination of its outstanding strength, biocompatibility, and tunable degradability [3,4].

A single strand of natural silk fiber contains two parallel SF fiber cores surrounded by a protective, glue-like sericin coating [5]. To fabricate SF-based products for utilization in textiles and biomedical materials, silk generally undergoes a critical degumming procedure to separate the SF from the sericin covering. In textiles, the presence of sericin creates a harsh and stiff feeling to the silk fibers, obscures a rich luster and whiteness, and prevents the elasticity of SF, subsequently, resulting in poor dyeing performance in terms of staining uniformity and color fastness because residual sericin prevents the dye and solution from penetrating during the wet treatment for silk [6]. In the field of SF-based biomaterials, removing sericin from the silk covering is essential to guarantee the biocompatibility of materials [7,8,9]. During the regeneration of SF, degumming accelerates the dissolution of SF fibers in a highly concentrated neutral salt solution, such as LiBr and CaCl_2_, since non-degummed fibers are resistant to solubilization [10]. Moreover, some research has suggested that the degumming process has a greater impact on the molecular weight (MW) of the regenerated SF than the dissolution process, although they both induce breakage of the peptide chains [11,12]. The MW is a fundamental factor that strongly affects the mechanical performance, biodegradation behavior, and thermal stability of the regenerated SF [13,14], and these issues are important in the design of SF-based biomaterials. Therefore, the degumming process has a substantial impact on the structure, properties, and application of SF-based materials. 

In conventional degumming processes, the silk fibers are treated with Na_2_CO_3_, soap, synthetic detergents, or organic acid solution [15,16,17]. The preferred commercially available degumming agents are based on Na_2_CO_3_ and Marseille soap because they have a high degumming efficiency and simple operation [18]. Sericin can be completely removed from silk when boiled in 0.02 M Na_2_CO_3_ for 60 min, but the breaking strength of the resulting silk fibers is significantly reduced by approximately 56% [19]. When boiled three times in 0.5 g/L Na_2_CO_3_ for 30 min and subsequently dissolved in LiBr aqueous solution, the MW of regenerated SF is mainly distributed below 100 kDa [18], which is far lower than the MW of native SF. Meanwhile, the falling in aesthetic properties, such as a dull appearance and poor handling, often occurs after degumming [20,21]. Therefore, Na_2_CO_3_ fully removed the sericin, but simultaneously caused destructive damage to SF. Commercially available Marseille soap is produced by the saponification of olive oil reacting with sodium hydroxide. The solution is generally weakly alkaline. Silk fibers would yield a 13.53% loss of strength when treated with a 10 g/L Marseille soap solution at 98 °C for 120 min [22]. Marseille soap is expensive and can be replaced by synthetic detergents in continuous degumming systems since it is incapable of buffering the acidity generated by the accumulation of sericin hydrolysis products in the bath [23,24]. Both Na_2_CO_3_ and Marseille soap can achieve a high silk degumming efficiency, however, SF is also highly sensitive to alkaline conditions. The complete removal of sericin is often accompanied by hydrolysis, which destroys the peptide bonds in the main chains of SF when it is exposed to strong alkaline treatments [10,12]. This leads to the undesirable deterioration in mechanical properties and a reduced MW of SF.

The enzymatic treatment of silk has received remarkable attention as an alternative for degumming because it is performed by applying proteolytic enzymes that selectively react with only specific parts of the silk to destroy the unwanted sericin and causes little hydrolytic damage to fibroin [25]. Alkaline proteases are considerable degumming reagents owing to their activity and stability under alkaline conditions where they can readily break the sericin chains and produce a higher degumming efficiency for silk [26]. In a previous study, silk fabric was treated with alkaline proteases 3374-L and GC 897-H derived from genetically modified *Bacillus subtilis* at pH 10 for 30 min; the degumming ratio was approximately 25% and 22% for the two proteases, respectively, which is close to the results of soap-alkali degumming [27,28]. It has also been demonstrated that the microbial alkaline proteases of Conidiobolus Brefeldianus and BOA-2, as well as the alkaline protease MTCC 5184 produced by *Beauveria* sp. all effectively removed sericin at pH 9.0–10.0, resulting in smooth and separated fibers without sericin deposits [29,30]. The combination of lipase and protease to treat silk at pH 8.5 resulted in the similar weight loss, a cleaner longitudinal surface, and increased wettability compared to the treatment with Marseille soap [31]. However, the presence of an alkaline environment increases the deterioration degree of silk fibers that primarily results in a sharp decline in the MW and mechanical properties of SF. Therefore, the application of a neutral protease that is highly specific to sericin and has high catalytic efficiency under neutral conditions will be a potential strategy for silk degumming.

SF is the basic fibrous protein of silk and has a highly repetitive-Gly-Ala-Gly-Ala-Gly-Ser-motif, which drives the formation of a large number of stable antiparallel β-sheet microcrystallites [32]. The highly oriented and crystalline structure is responsible for the strong resistance and stability of fibroin in most solvents, including water, ethanol, dilute acids, and dilute bases. In contrast to SF, sericin is a water-soluble globular protein that contains serine, aspartate, and glycine, totaling over 60% of the overall composition. Approximately 70% of the side groups are hydrophilic, such as hydroxyl, carboxyl, and amino groups, endowing sericin with better water absorption and solubility than SF, which comprises 79% hydrophobic residues [17,33]. The primarily amorphous structure is dominated by random coil that is easily penetrated by degumming reagents [34]. The amino acid composition and molecular conformation make sericin more susceptible to being hydrolyzed and removed by extensive degumming methods. Importantly, SF and sericin also differ greatly in their content of some amino acids. For example, in sericin, L-arginine and L-lysine occupy approximately 4.2% and 5.5%, while in fibroin they account for a relatively lower percentage of approximately 0.90% and 0.45%, respectively [35]. This significant difference in amino acid composition may provide a valuable information for the removal of sericin by enzymatic degumming. 

Papain is a neutral proteolytic enzyme that shows extensive proteolytic activity towards proteins and peptides, and functions over a wide pH range of 3.0 to 9.5 [36,37]. It preferentially cleaves peptide bonds between L-arginine or L-lysine residue and another amino acid residue [38,39], L-arginine and L-lysine are much more abundant in sericin than in fibroin. Previous studies have showed that degumming with papain can reduce the damage to SF due to the absence of alkaline reagents [40,41,42]. Therefore, papain is expected to specifically cleave sericin due to the substantial differences in the content of susceptible targets between sericin and SF and is regarded as a potential and promising choice for silk degumming.

We envisaged that sericin could be fully removed by applying a papain solution without a reducing agent or surfactant under nearly neutral conditions based on the significant difference in amino acid content between sericin and fibroin. The papain catalysis enabled the harsh and stiff sericin to be gradually hydrolyzed and dissolved from the surface of the silk through the fixed-point cleavage of specific amino acid reaction sites. Due to the low content of reactive sites at L-arginine and L-lysine residues in SF, the enzymatically degummed silk sustained less hydrolytic damage to the SF chains even though the sericin was completely isolated. These benefits led to an obviously increased mechanical strength and MW of SF compared with the traditional Na_2_CO_3_ degumming method.

The aim of this study was to prepare completely degummed and high MW SF by applying papain to specifically degrade sericin. First, we investigated the influence of papain concentration on the degumming efficiency of silk fiber by assessing the percent weight loss, the color of picric acid and carmine staining (PACS), the K/S value, and surface morphology to obtain the optimal papain concentration. The traditional Na_2_CO_3_ degumming method was also performed for comparison. Next, tensile strength tests, amino acid analysis, and sodium dodecyl sulfate-polyacrylamide gel electrophoresis (SDS-PAGE) of the resultant sericin-free silk fibers were conducted to evaluate the effect of the degumming process on the mechanical properties, amino acid composition, and MW of the resulting silk fibroin.

## 2. Materials and Methods

### 2.1. Materials

Grade 6A *Bombyx mori* raw silk (reeled silk fiber) with a 20/22 denier was used for this work (supplied by Haian Soho International Co., Ltd., Nantong, China). Papain (EC3.4.22.2, 8 × 10^5^ units/g solid, lyophilized powder) was purchased from Beijing Solarbio Science and Technology Co., Ltd. (Beijing, China). Carmine was obtained from Shanghai Chemical Reagent Co., Ltd. (Shanghai, China). Picric acid was purchased from Zhejiang Taizhou Chemical Industry Co., Ltd. (Taizhou, China). Ammonium solution (25 wt %) was purchased from Sinopharm Chemical Reagent Co., Ltd. (Shanghai, China). The 7-day-old fifth instar larvae of *Bombyx mori* silkworm were provided by the Rudong Guidance Station of Sericulture (Nantong, China). Cellulose dialysis membranes, 12–14 kDa, were obtained from Pierce (Waltham, MA, USA). All other chemicals were of analytical grade except as specified. Deionized water was used throughout all experiments. 

### 2.2. Silk Degumming with Different Concentrations of Papain 

The divided raw silk fibers were degummed at 85 ± 2 °C for 60 min in papain solution of various concentrations (0.01, 0.05, 0.1, 0.3, 0.5, 1.0, 3.0, 4.0, 6.0 g/L) with a bath ratio of 1:50. The pH value was adjusted to 6.0 and maintained throughout the process by the addition of pH 6.0 sodium dihydrogen phosphate-citric acid buffer. The degummed silks were immediately placed into boiling water to inactivate the enzymes and then thoroughly washed with warm distilled water. Traditional degumming by the Na_2_CO_3_ method was used as a control and carried out according to previously reported protocols [43]. In brief, raw silk fibers were boiled three times for 30 min each in 0.5 g/L Na_2_CO_3_ aqueous solution and then thoroughly rinsed with deionized water. Next, all degummed silk fibers were air-dried in an oven at 60 ± 2 °C. The degummed silk fibers were marked as X g/L papain-silk and Na_2_CO_3_-silk for a simple description according to the applied degumming method, where X represents the applied enzyme concentration.

### 2.3. Weight Loss Measurements 

The degumming ratio D_r_ (%) was used to evaluate the degree of sericin removed by the degumming treatment. It was expressed in terms of percentage weight loss from the samples after degumming (*n* = 5), according to Equation (1):

(1) $$D_{r}\;(\%)=\frac{W_{0}-W_{1}}{W_{0}}\times 100$$

where W_0_ (g) and W_1_ (g) are the conditioned weights of silk fibers before and after degumming, respectively.

Firstly, the moisture regain H (%) of the sample taken from raw silk before degumming was determined by weight loss after drying at 140 ± 2 °C to reach a constant weight. Then, the silk to be degummed was weighed and recorded as W_0_’ (g), the conditioned weight W_0_ was expressed as the weight of silk at commercial moisture regain (11%) according to Equation (2):

(2) $$W_{0}=\frac{W_{0}{}^{\prime }}{1+\frac{H}{100}}\times (1+\frac{11}{100})$$

After degumming, all silk samples were dried in an oven at 140 ± 2 °C until reaching a constant weight, marked as W_1_’(g). The conditioned weight W_1_ was expressed as the weight of silk at commercial moisture regain (11%) according to Equation (3):

(3) $$W_{1}{=W}_{1}{}^{\prime }\times (1+\frac{11}{100})$$

In order to describe the residue degree of sericin intuitively, degumming with Na_2_CO_3_ was taken as the standard 100% weight loss; the degumming ratio was marked as D_s_ (%) accordingly. The residual sericin ratio SR (%) of the papain-silk samples was calculated by comparing the enzyme treatment with the Na_2_CO_3_ method, the SR (%) was expressed by Equation (4):

(4) $$\mathrm{Residual}\;\mathrm{sericin}\;\mathrm{ratio}\;\mathrm{SR}\;(\%)=D_{s}\;(\%)-D_{e}\;(\%)$$

where D_s_ (%) and D_e_ (%) are the degumming ratio of silk treated by 0.5 g/L Na_2_CO_3_ and treated by the papain solution with various concentrations, respectively.

### 2.4. Picric Acid and Carmine Staining (PACS) Assay

The degumming efficiency was qualitatively evaluated by the PACS assay. The staining solution was prepared as follows. Briefly, 1 g of carmine was dissolved in 10 mL of 25 wt % ammonia, and heated at 45 °C after the addition of 20 mL of deionized water. After the solution was cooled, 45 mL of saturated picric acid solution was added before bringing to a final volume of 100 mL with distilled water and adjusting the pH to 8.0–9.0 by adding 0.1 mol/L HCl. The dried degummed silk samples and the non-degummed raw silk were immersed in the staining solution for 5 min and then washed several times with distilled water and dried at 60 °C. The stained silks were examined and photographed for evaluation.

The ratio of the absorption and scattering coefficient (K/S) value of the stained silks was measured with a HunterLab UltraScan PRO reflectance spectrophotometer (Standard Illuminant D65; 10° Standard Observer, Reston, VA, USA) over the wavelength range of 350 to 700 nm (*n* = 5 for each sample).

### 2.5. Morphological Characterization of Degummed Silks

The morphology of the degummed silk samples and the non-degummed raw silk fibers (blank control) was observed using field-emission scanning electron microscopy (SEM, S-4800, Hitachi, Tokyo, Japan) after being sputtered with gold.

### 2.6. Tensile Properties

The fineness of the silk samples was determined by measuring the length and mass of the filaments. An Instron 3365 material testing machine (Norwood, MA, USA) equipped with a 10 N load cell was used to measure the mechanical properties of single fibers fixed to a paper frame. The samples were preconditioned at 20 ± 2 °C and 65 ± 5% RH for 24 h prior to measurement. A total of 20 single fibers for each sample group were tested at a drawing speed of 10 mm/min with a gauge length of 20 mm. The results of raw silk fibers and Na_2_CO_3_-silk were used as control groups for comparison with 3.0 g/L papain-silk. The results of mechanical properties were expressed as the mean ± standard deviation (SD) of each group and statistically analyzed using the Student’s *t*-test [44]. For each test, a probability value of *p* < 0.05 was considered to be statistically significant.

### 2.7. Preparation of Regenerated SF Solution and SF Solids 

The 3.0 g/L papain-silk was dissolved in 9.3 M LiBr solution with a bath ratio of 1:30 at 60 ± 2 °C for 1 h. The mixture was then dialyzed in deionized water for three days until an AgNO_3_ test could detect no trace of bromide ions. The resulting solution was filtered and centrifuged at 5000 rpm for 10 min at 4 °C to remove aggregates and impurities. As a control group, a Na_2_CO_3_-derived SF solution was prepared by the same procedures as above from the Na_2_CO_3_-degummed silk. The regenerated SF solution were labeled as 3.0 g/L papain-SF and Na_2_CO_3_-SF, respectively.

A native SF sample extracted from the silk glands of *Bombyx mori* silkworms was set as another control group (marked as Gland-SF). The silk glands were harvested from the abdominal side of seven-day-old fifth instar larvae of the *Bombyx mori* silkworm and then washed several times in 0.7% ice-cold normal saline. The gel-like fibroin samples were collected from the posterior division of the silk gland and gradually dissolved in double-distilled water at 4 °C; the SF solution was then filtered. The obtained Gland-SF solution and a portion of the 3.0 g/L papain-SF and Na_2_CO_3_-SF solution were freeze-dried for amino acid analysis.

### 2.8. Amino Acid Analysis 

An amino acid analysis was carried out on a Hitachi L-8900 Amino Acid Analyzer. The SF solids were hydrolyzed in 6 N HCl for 24 h at 110 °C. After removing the HCl, the concentration of the residues was diluted to 0.02% with 0.02 N HCl and filtered with Millipore 0.22-μm syringe filters (Milford, CT, USA) [45].

The amino acid composition deduced from the genomic sequence of *Bombyx mori* silk fibroin (Genomic-SF) was taken as another control group [46]. 

### 2.9. Sodium Dodecyl Sulfate-Polyacrylamide Gel Electrophoresis (SDS-PAGE)

The molecular weight distribution (MWD) of the regenerated 3.0 g/L papain-SF and Na_2_CO_3_-SF solution was determined by SDS-PAGE [45]. The concentration of the stacking gel was 5%, and the concentrations of the separating gels were 6% and 15%. Pre-stained protein served as the MW markers (5–100 and 50–250 kDa) for examining the MW values. The gels were stained with an Easy Stain Coomassie Blue Kit (Invitrogen, Carlsbad, CA, USA). 

The MW of target bands was further quantified by scanning densitometry, and the gray intensity values of the protein bands were measured with the NIH ImageJ software. This involved selecting target regions and determining the mean gray value. The band intensity was expressed relative to the density of the band in the control sample [47].

## 3. Results and Discussion

### 3.1. Degumming Ratio

**Figure 1:**
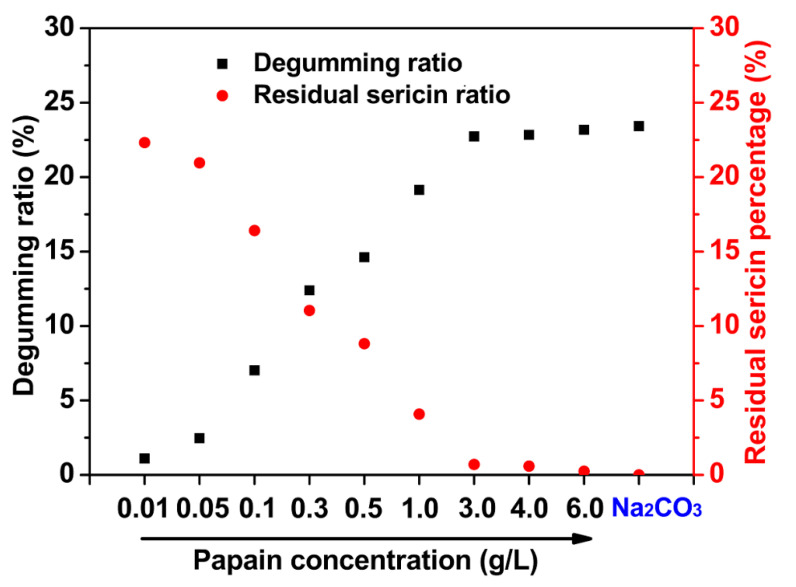
Degumming ratio and residual sericin percentage of silk fibers degummed with different concentrations of papain and traditional 0.5 g/L Na_2_CO_3_ as a control. The error bars are not visible because they are smaller than the marker size.

In this study, a nearly neutral condition (pH = 6.0) was chosen for the degumming system to avoid creating an alkaline environment and to guarantee high catalytic activity of papain. The degumming ratio and the degree of residual sericin on the silk fibers subjected to different concentrations of papain were determined. The results were compared with those of the traditional Na_2_CO_3_ degumming method (control group). As seen from Figure 1, the weight loss of the silk samples gradually increased from 1.11 ± 0.06% to 22.73 ± 0.08% with the concentration of papain increasing from 0.01 to 3.0 g/L and then the value hardly increased, at about 23% when papain concentrations were >3.0 g/L, which was similar to the degumming ratio of the traditional Na_2_CO_3_ method (23.43 ± 0.04%). The residual sericin remaining on the silk fibers correspondingly declined to a low level with increasing papain concentrations, indicating the effective removal of sericin. The enzyme concentration is a key factor affecting the efficiency of catalytic hydrolysis. To some degree, an increasing concentration provides more bonding opportunities between the enzyme and substrates at the reaction sites. An increase in the papain concentration accelerated the stripping of sericin. Since papain is highly specific to sericin, it might cause little SF degradation, while sericin was completely removed. The degummed ratios did not change significantly even as the enzyme concentration was increased from 3.0 to 6.0 g/L. Therefore, 3.0 g/L was considered as the critical concentration for the complete separation of SF and sericin.

### 3.2. PACS Assay

**Figure 2:**
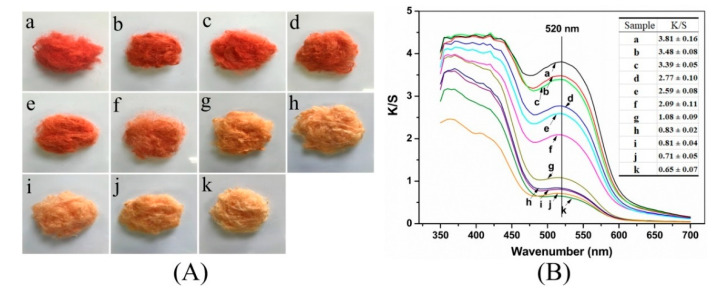
(**A**) Color change and (**B**) the absorption and scattering coefficient (K/S) value of silk samples after picric acid and carmine staining: (**a**) Non-degummed raw silk, (**b**–**j**) degummed with 0.01, 0.05, 0.1, 0.3, 0.5, 1.0, 3.0, 4.0, and 6.0 g/L papain, respectively, (**k**) degummed with 0.5 g/L Na_2_CO_3_.

PACS assay was used to estimate the effectiveness of silk fibers degumming by papain. Fibroin selectively adsorbs picric acid molecules in alkaline conditions, generating a yellow color, whereas both carmine and picric acid are able to adhere to sericin simultaneously because of its stronger adsorptive properties. When sericin is present on silk fibers, the silk is stained red because the red color is more visible than the yellow color [48,49]. Therefore, the depth of the red color indirectly reflects the amount of retained sericin. If the silk turns yellow, this suggests that all sericin has been removed. PACS assay has been recognized as a typical and standard method to identify the complete removal of sericin. Figure 2A(a) shows that the non-degummed raw silk was stained dark red due to the presence of large amounts of sericin coating on silk fibers. Conversely, the Na_2_CO_3_-silk displayed a yellow color and exhibited a shiny appearance and soft texture instead of the stiff and dull properties of raw silk, implying that the sericin had been removed (Figure 2A(k)). When the concentration of papain solution was gradually increased, the red color gradually faded and even disappeared. The yellow color visible on the surface suggested that the residual sericin content had decreased. The red color was dominant when the concentration of papain solution ranged from 0.01 to 0.5 g/L, which resulted from the sericin remaining on the fibers (Figure 2A(b–f)). When the enzyme concentration exceeded 1.0 g/L, the treated silks emerged with a light yellow color similar to that of the Na_2_CO_3_-silk (Figure 2A(h–j)), however, it is difficult to directly assess the degree of degumming solely by the differences in color.

The K/S value at 520 nm representing a red-absorbing wavelength was detected to assess the residual degree of sericin. As shown in Figure 2B, the raw silk showed the highest K/S value of 3.81; the value was the lowest for Na_2_CO_3_-silk, at 0.65. The K/S values of the silks that underwent papain treatment also showed a gradually decreasing trend as the papain concentration was elevated. The K/S values of silk samples at ≥ 3.0 g/L papain were close to that of Na_2_CO_3_-silk, indicating that nearly all of the sericin was removed after degumming (Figure 2B(h–j)). These results corresponded with the obtained weight loss results shown in Figure 1, which jointly demonstrated that 3.0 g/L of papain may be enough for complete degumming.

### 3.3. Morphological Observation of Degummed Silk Fibers

**Figure 3:**
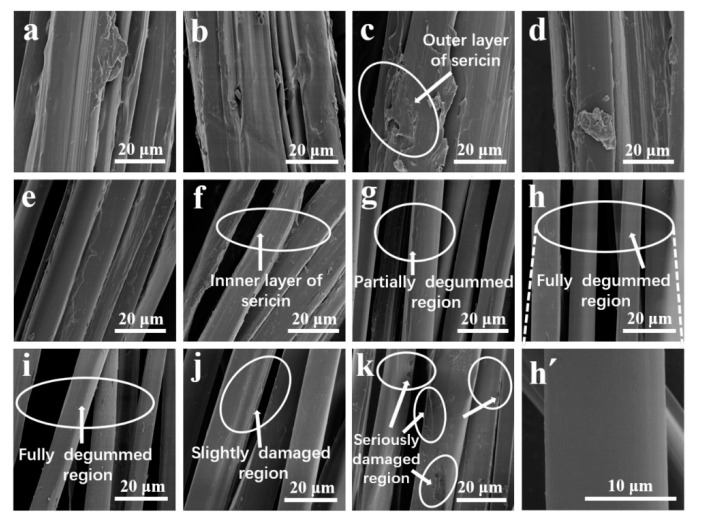
SEM images of silk fiber surfaces. (**a**) Non-degummed raw silk fibers, (**b–j**) degummed with 0.01, 0.05, 0.1, 0.3, 0.5, 1.0, 3.0, 4.0, and 6.0 g/L papain, (**k**) degummed with 0.5 g/L Na_2_CO_3_, (**h’**) magnified image of (**h**). Scale bars: (**a**–**k**) 20 μm, (**h’**) 10 μm.

SEM observation revealed that the silk fibers before degumming were covered with a relatively large amount of sericin and acted as a binder that could stick to fibroin, as illustrated in Figure 3a. With increasing papain concentrations, the peripheral sericin layers gradually broke away from the core fibroin fiber, and the longitudinal outline of the silk fiber was revealed (Figure 3b–g). The 3.0 g/L papain-silk showed a highly uniform, clean, and smooth fibroin surface, indicating that the sericin was almost completely removed. The degummed fibers were approximately 9–11.5 μm in width (Figure 3h,h’), which is in the expected range of native single SF fiber [50]. However, undesired destruction and damage to the silk fibroin occurred at 4.0 and 6.0 g/L papain (Figure 3i,j), which was attributed to slight hydrolysis of SF after the complete exfoliation of sericin. The surface of the Na_2_CO_3_-silk had no sericin attached, but it suffered extensive damage, with deeper grooves and larger cracks (Figure 3k). This finding shows that Na_2_CO_3_ acts unselectively towards the fibers and penetrates the silk fiber to hydrolyze the fibroin core, resulting in physical damage and deteriorating the quality of fibroin fiber [11,14,18].

The SEM results strongly suggested the occurrence of excessive degumming at > 3.0 g/L papain and with conventional 0.5 g/L Na_2_CO_3_. Combined with the obtained weight loss and PACS results, it was showed that degumming with 4.0 and 6.0 g/L papain resulted in the complete removal of sericin and a slight hydrolysis and degradation of SF. The slight damage to silk fibroin also indicated that the specificity enabled by papain degumming constituted a milder and more effective approach than the Na_2_CO_3_ treatment. A concentration of 3.0 g/L papain was found to be the critical concentration at which sericin could be entirely broken down without obvious damage and deterioration of the fibroin protein. With the aim of producing highly purified SF, the mildest degumming concentration of 3.0 g/L papain was chosen for subsequent experiments.

### 3.4. Effect of Degumming Method on the Mechanical Performance of Silk Fibers

**Figure 4:**
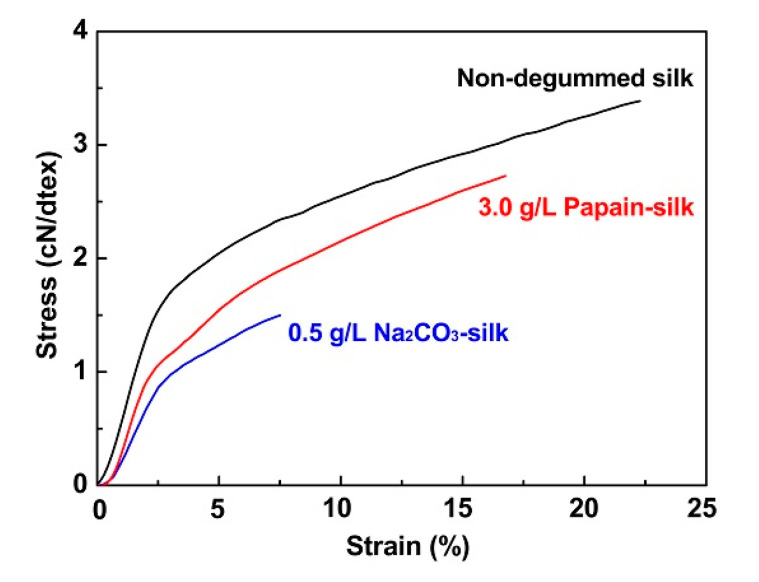
Stress-strain curves of sericin-free silk fibers prepared by two degumming methods, with non-degummed raw silk as a control.

Tensile properties are important parameters by which to evaluate fiber performance after degumming. Harsh degumming conditions cause substantial bond breakage, protein chain degradation, and serious microstructure destruction of SF, which acts as the core for bearing force. This degradation may induce deterioration of the mechanical performance of the SF fibers. Tensile tests were carried out to evaluate the mechanical properties of sericin-free fibers after different degumming methods. The stress-strain curves of the fibers are presented in Figure 4. The maximum tensile strength, elongation at break, and Young’s modulus are displayed in Table 1. 

The highest tensile strength and strain of silk fibers before degumming was 3.4 ± 0.3 cN/dtex and 25.2 ± 4.7%, respectively; the highest Young’s modulus value was 0.8 ± 0.1 cN/dtex. These data indicate the impressive strength of SF, which arises from its ordered hierarchical structure and β-sheet crystallites containing substantial intramolecular/intermolecular hydrogen bonds together with intersheet Van der Waals and hydrophobic interactions [32,51]. Additionally, sericin serves as an adhesive that hinders slippage between filaments and reinforces the fiber strength [23]. When Na_2_CO_3_ is used as a degumming agent, the temperature of the Na_2_CO_3_ solution has a great influence on the strength of the silk fiber after degumming [19,52,53]. In this study, the silk fibers were degummed with a boiled Na_2_CO_3_ aqueous solution. The strength of Na_2_CO_3_-silk fiber sharply decreased to 1.5 ± 0.2 cN/dtex, with the lowest Young’s modulus of 0.4 ± 0.1 cN/dtex, demonstrating a considerable decline in mechanical properties. This was mainly attributed to the breakage and scission of the SF chains and the partial hydrolysis of silk macromolecules during the treatment [24]. Defects exposed on the surface of SF are potentially weak regions that can readily break down when external force is applied. This was demonstrated by the lower strain of 8.7 ± 2.2% for the Na_2_CO_3_-silk fibers. As expected, the 3.0 g/L papain-silk fiber showed higher tensile strength (2.7 ± 0.2 cN/dtex) and noticeably increased strain (16.5 ± 2.2%) and Young’s modulus (0.7 ± 0.1 N/dtex) compared to the Na_2_CO_3_-silk fiber, confirming the less destructive exfoliation of the SF component and more limited impact on the mechanical properties. The results indicated that the mild and efficient degumming produced by the 3.0 g/L papain solution induced less degradation of the SF chains while simultaneously is available to achieve sericin-free silk fibers with better mechanical performances.

### 3.5. Amino Acid Analysis

**Figure 5:**
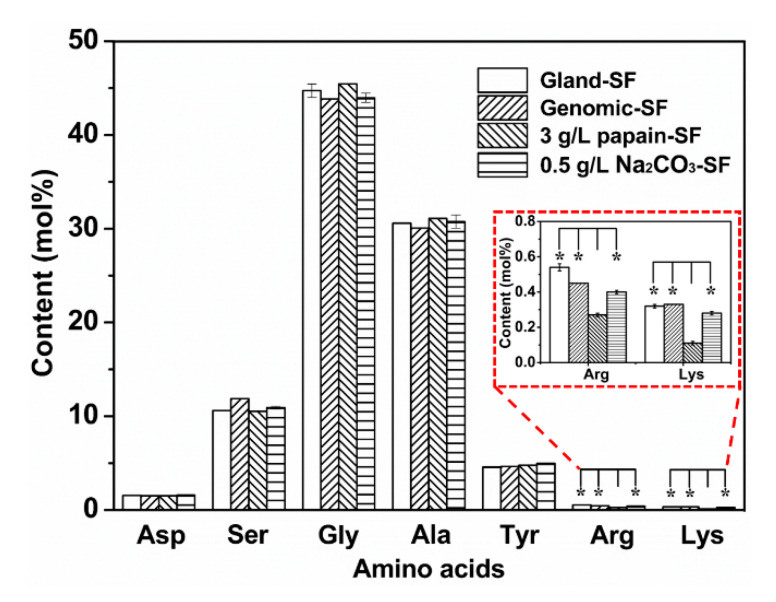
The contents of the main amino acids in silk fibroin obtained by the different methods. * Indicates significant differences compared with 3.0 g/L papain-SF at *p* < 0.05.

Amino acid analysis was conducted to compare the amino acid composition of Gland-SF, Genomic-SF, 3.0 g/L papain-SF, and 0.5 g/L Na_2_CO_3_-SF. Figure 5 shows that Ser, Gly, and Ala accounted for approximately 87% of the total amino acids present in 3.0 g/L papain-SF, which has no significant difference with the results derived from Genomic-SF and Gland-SF. No significant differences in the major components implied that papain degumming had no effect on the crystallization regions of SF. However, the amount of Lys and Arg was far lower in 3.0 g/L papain-SF than that in other groups, for example, compared with natural Gland-SF (0.11 vs. 0.33 mol% and 0.27 vs. 0.45 mol%, respectively, *p* < 0.05). These obvious differences revealed that Arg and Lys were characteristic cleavage sites degraded by papain. Although the sericin was removed, the process did not cause excessive SF degradation because the hydrolysis of SF was initiated by the breakage of Arg and Lys without detectable loss of the other amino acids. The decreased relative content of Arg and Lys in SF also demonstrated the specificity of papain to sericin, which has a much higher content of Lys and Arg than SF. These results further implied that the SF obtained from 3.0 g/L papain retains a high level of peptide chain integrity.

### 3.6. Influence of Degumming Methods on the MWD

**Figure 6:**
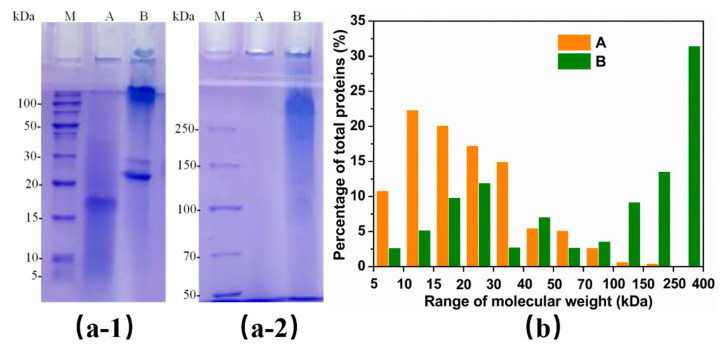
(**a-1**) and (**a-2**) SDS-PAGE patterns of regenerated SF derived from different degumming methods. (**M**) Molecular weight marker, (**A**) Na_2_CO_3_-SF, (**B**) 3.0 g/L papain-SF. Separating gel concentration: (**a-1**) 15%, (**a-2**) 6%. (**b**) Semi-quantitative statistics of molecular weight distributions (MWDs) based on SDS-PAGE patterns.

The MWD of the regenerated SF solution were measured by SDS-PAGE. *Bombyx mori* silk fibroin is mainly composed of six heavy chains (~350 kDa) and six light chains (~25.8 kDa) linked by a single disulfide bond [10,50]. Alkaline degumming can result in severe degradation of SF macromolecules by breaking the peptide bonds in the main chains, which lowers the MW of the regenerated SF [54,55]. In the SDS-PAGE results shown in Figure 6(a-1),(a-2), the Na_2_CO_3_-SF had a broad MWD in the lower MW ranges, with sequential bands from 5 to 100 kDa. Notably, 3.0 g/L papain-SF exhibited higher MW bands distributed ranging from 100 to 250 kDa. A prominent band at ~25 kDa could be assigned to the L-chain in sample B, while this band was not present in sample A, indicating that the SF degradation by 3.0 g/L papain is milder.

The semi-quantitative analysis of the MWD of the samples was accomplished by determining the gray intensity values in the different MW regions. As shown in Figure 6b, 3.0 g/L papain-SF had a significantly higher percentage of polypeptides with a MW > 100 kDa compared to Na_2_CO_3_-SF, which illustrated the presence of longer SF chains after the papain treatment. The statistical results revealed an average MW of approximately 60 kDa for Na_2_CO_3_-SF, while the average MW of papain-SF was nearly 145 kDa. These results proved that the 3.0 g/L papain-silk underwent a milder sericin removal process and generated higher MW SF compared with the traditional 0.5 g/L Na_2_CO_3_ method.

### 3.7. Potential Degumming Mechanism

During the degumming process, only sericin is desired to be removed from the core-shell composite structure of silk fibers, with little damage to the fibroin. In the traditional degumming method, the Na_2_CO_3_ solution is strongly alkaline owing to the characteristics of strong base-weak acid salt. Peptide bonds in the main chains of proteins are easily attacked under alkaline environment and the nonspecific reaction caused the hydrolysis of the peptide chains [56], which is the main reason for the removal of sericin. The solubility of sericin can be also enhanced by changing the –COOH on the side chain of sericin to –COONa^+^ [57]. Fully swollen silk fibers become gradually permeable to small molecules, and Na_2_CO_3_ can be easily adsorbed onto the core fibroin, provoking undesired hydrolysis of fibroin. In addition, when Na^+^ is combined with SF, a lot of water molecules will be brought and penetrated into amorphous regions and act as a powerful plasticizer to disrupt the inter- and intra-molecular hydrogen bond due to the strong hydratability of Na^+^, the destruction of hydrogen bond further reduces the van der Waals force between the protein chains, resulting in increased possibility for relative displacements of protein chains and stress relaxation [58,59]. Although sericin can be completely removed with the aid of Na_2_CO_3_, it remains to cause serious breakage to the SF molecular chains. This damage can lead to a false degumming ratio that includes the weight loss arising from the degraded SF and results in the severe loss of tensile strength and the decrease in MW of SF, which has been previously reported [18,19]. Compared with traditional degumming methods, enzymatic degumming has higher sericin specificity and milder reaction conditions. Considerable evidence for alkaline protease degumming has shown its advantages over Na_2_CO_3_ degumming. Nevertheless, the treatment under alkaline environment is harmful to silk fibers because silk has poor alkaline resistance. Therefore, in the current study, papain was adopted for silk degumming under nearly neutral conditions to avoid the use of alkaline conditions based on the substantial differences in amino acid composition between SF and sericin.

**Figure 7:**
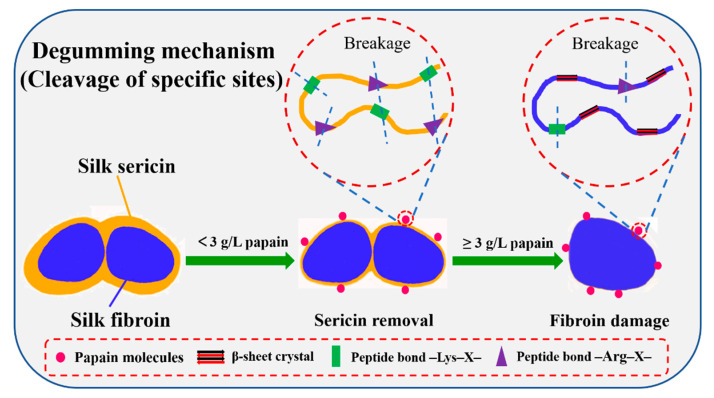
Underlying degumming mechanism of papain treatment.

The underlying degumming mechanism of papain treatment is shown in Figure 7. Due to the high MW (≈23 kDa) of the papain molecules, the presence of steric hindrance makes it difficult for the enzyme to penetrate into silk fibers. The degumming process is essentially considered as the layer-by-layer exfoliation of the sericin from the surface of silk fiber. Importantly, papain molecules specifically hydrolyze amino acid sequences of sericin by breaking the peptide bonds formed by L-arginine or L-lysine and another amino acid. As the sericin macromolecular chains are hydrolyzed and cracked, they are gradually exfoliated from the fiber surface layer-by-layer. The concentration of 3.0 g/L papain is adequate for the complete removal of sericin. When the papain concentration was increased above this, the SF became the target of papain due to a lack of sericin protection. The limited cleavage sites of SF leads to less SF damage, resulting in improved tensile strength and higher MW SF compared with traditional Na_2_CO_3_ degumming. Notably, the mild papain degumming method has a lower environmental impact, lower energy consumption, and can avoid residual alkaline regents in degummed products, all of which are powerful advantages that should be taken into account in industrial production.

## 4. Conclusions

The applicability and impact of using papain to degum *Bombyx mori* silk fibers was investigated. The effects of the enzymatic reaction on the degumming efficiency, mechanical properties, amino acid composition, and molecular weight were compared to those of the conventional Na_2_CO_3_ degumming method. The results showed that a papain concentration of 3.0 g/L was sufficient to achieve the complete removal of sericin without obvious damage to the silk fibroin owing to the specificity of papain for sericin. The resulting fibroin exhibited a higher molecular weight and an enhanced tensile performance. This study provides a novel degumming method with high efficiency and low environmental pollution for obtaining high molecular weight silk fibroin and contributes to improving the quality of SF-based materials.

## Figures and Tables

**Table 1:** Mechanical properties of silk fibers treated by two degumming methods (*n* = 20).

Sample	Breaking Stress (cN/dtex)	Breaking Strain (%)	Young’s Modulus (cN/dtex)
Non-degummed silk	3.4 ± 0.3	25.2 ± 4.7	0.8 ± 0.1
3.0 g/L papain-silk	2.7 ± 0.2 **	16.5 ± 2.2 *	0.7 ± 0.1 *
0.5 g/L Na_2_CO_3_-silk	1.5 ± 0.2	8.7 ± 2.2	0.4 ± 0.1

*, ** Indicates significant differences compared with Na_2_CO_3_-Silk at *p* < 0.05 and *p* < 0.01, respectively.

## References

[1] Cranford S.W., Tarakanova A., Pugno N.M., Buehler M.J. (2012). Nonlinear material behaviour of spider silk yields robust webs. Nature.

[2] Wang C., Li X., Gao E., Jian M., Xia K., Wang Q., Xu Z., Ren T., Zhang Y. (2016). Carbonized silk fabric for ultrastretchable, highly sensitive, and wearable strain sensors. Adv. Mater..

[3] Omenetto F.G., Kaplan D.L. (2010). New opportunities for an ancient material. Science.

[4] Rockwood D.N., Preda R.C., Yucel T., Wang X., Lovett M.L., Kaplan D.L. (2011). Materials fabrication from *Bombyx mori* silk fibroin. Nat. Protoc..

[5] Vollrath F., Porter D. (2009). Silks as ancient models for modern polymers. Polymer.

[6] Mahmoodi N.M., Arami M. (2009). Numerical finite volume modeling of dye decolorization using immobilized titania nanophotocatalysis. Chem. Eng. J..

[7] Thurber A.E., Omenetto F.G., Kaplan D.L. (2015). In vivo bioresponses to silk proteins. Biomaterials.

[8] Altman G.H., Diaz F., Jakuba C., Calabro T., Horan R.L., Chen J.S., Lu H., Richmond J., Kaplan D.L. (2003). Silk-based biomaterials. Biomaterials.

[9] Teuschl A.H., Van Griensven M., Redl H. (2014). Sericin removal from raw *Bombyx mori* silk scaffolds of high hierarchical order. Tissue Eng. Part C Methods.

[10] Wray L.S., Hu X., Gallego J., Georgakoudi I., Omenetto F.G., Schmidt D., Kaplan D.L. (2011). Effect of processing on silk-based biomaterials: Reproducibility and biocompatibility. J. Biomed. Mater. Res. B Appl. Biomater..

[11] Wang Q., Chen Q., Yang Y., Shao Z. (2013). Effect of various dissolution systems on the molecular weight of regenerated silk fibroin. Biomacromolecules.

[12] Kim H.J., Kim M.K., Lee K.H., Nho S.K., Han M.S., Um I.C. (2017). Effect of degumming methods on structural characteristics and properties of regenerated silk. Int. J. Biol. Macromol..

[13] Cho H.J., Ki C.S., Oh H., Lee K.H., Um I.C. (2012). Molecular weight distribution and solution properties of silk fibroins with different dissolution conditions. Int. J. Biol. Macromol..

[14] Kim H.H., Song D.W., Kim M.J., Ryu S.J., Um I.C., Ki C.S., Park Y.H. (2016). Effect of silk fibroin molecular weight on physical property of silk hydrogel. Polymer.

[15] Zhang F., Lu Q., Ming J., Dou H., Liu Z., Zuo B., Qin M., Li F., Kaplan D.L., Zhang X. (2014). Silk dissolution and regeneration at the nanofibril scale. J. Mater. Chem. B.

[16] Gupta D., Agrawal A., Chaudhary H., Gulrajani M., Gupta C. (2013). Cleaner process for extraction of sericin using infrared. J. Clean. Prod..

[17] Moazami A., Montazer M., Rashidi A., Rahimi M.K. (2010). Antibacterial properties of raw and degummed silk with nanosilver in various conditions. J. Appl. Polym. Sci..

[18] Dou H., Zuo B. (2014). Effect of sodium carbonate concentrations on the degumming and regeneration process of silk fibroin. J. Text. Inst..

[19] Nultsch K., Bast L.K., Näf M., Yakhlifi S.E., Bruns N., Germershaus O. (2018). Effects of silk degumming process on physicochemical, tensile, and optical properties of regenerated silk fibroin. Macromol. Mater. Eng..

[20] Pantano M.F., Berardo A., Pugno N.M. (2016). Tightening slip knots in raw and degummed silk to increase toughness without losing strength. Sci. Rep..

[21] Pérez-Rigueiro J., Elices M., Llorca J., Viney C. (2002). Effect of degumming on the tensile properties of silkworm (*Bombyx mori*) silk fiber. J. Appl. Polym. Sci..

[22] Wang F., Cao T., Zhang Y. (2015). Effect of silk protein surfactant on silk degumming and its properties. Mater. Sci. Eng. C.

[23] Ho M.P., Wang H., Lau K.T., Lee J.H., Hui D. (2012). Interfacial bonding and degumming effects on silk fibre/polymer biocomposites. Compos. Part B Eng..

[24] Ho M.P., Wang H., Lau K. (2012). Effect of degumming time on silkworm silk fibre for biodegradable polymer composites. Appl. Surf. Sci..

[25] Arami M., Rahimi S., Mivehie L., Mazaheri F., Mahmoodi N.M. (2007). Degumming of persian silk with mixed proteolytic enzymes. J. Appl. Polym. Sci..

[26] Suwannaphan S., Fufeungsombut E., Promboon A., Chim-Anage P. (2017). A serine protease from newly isolated Bacillus sp. for efficient silk degumming, sericin degrading and colour bleaching activities. Int. Biodeter. Biodegr..

[27] Kim J., Kwon M., Kim S. (2016). Biological degumming of silk fabrics with proteolytic enzymes. J. Nat. Fibers.

[28] Freddi G., Mossotti R., Innocenti R. (2003). Degumming of silk fabric with several proteases. J. Biotechnol..

[29] More S.V., Khandelwal H.B., Joseph M.A., Laxman R.S. (2013). Enzymatic degumming of silk with microbial proteases. J. Nat. Fibers.

[30] More S.V., Chavan S., Prabhune A.A. (2017). Silk degumming and utilization of silk sericin by hydrolysis using alkaline protease from *Beauveria Sp.* (MTCC 5184): A green approach. J. Nat. Fibers.

[31] Gulrajani M.L., Agarwal R., Grover A., Suri M. (2000). Degumming of silk with lipase and protease. Indian J. Fibre. Text. Res..

[32] Keten S., Xu Z., Ihle B., Buehler M.J. (2010). Nanoconfinement controls stiffness, strength and mechanical toughness of beta-sheet crystals in silk. Nat. Mater..

[33] Braun F. (2003). Modelling self assembly of natural silk solution. Int. J. Biol. Macromol..

[34] Kundu S.C., Dash B.C., Dash R., Kaplan D.L. (2008). Natural protective glue protein, sericin bioengineered by silkworms: Potential for biomedical and biotechnological applications. Prog. Polym. Sci..

[35] Ude A.U., Eshkoor R.A., Zulkifili R., Ariffin A.K., Dzuraidah A.W., Azhari C.H. (2014). *Bombyx mori* silk fibre and its composite: A review of contemporary developments. Mater. Design..

[36] Alpay P., Uygun D.A. (2015). Usage of immobilized papain for enzymatic hydrolysis of proteins. J. Mol. Catal. B Enzym..

[37] Azmi N.H.M. (2010). The Extraction of Amino Acids from Papaya Leaves using Supercritical Carbon Dioxide with Water. Ph.D. Thesis.

[38] Lowe G. (1970). The structure and mechanism of action of papain. Philos. Trans. R. Soc. Lond. B Biol. Sci..

[39] Bayramoglu G., Senkal B.F., Yilmaz M., Arica M.Y. (2011). Immobilization and stabilization of papain on poly(hydroxyethyl methacrylate-ethylenglycol dimethacrylate) beads grafted with epoxy functional polymer chains via surface-initiated-atom transfer radical polymerization (SI-ATRP). Bioresour. Technol..

[40] Vyas S.K., Shukla S.R. (2015). Comparative study of degumming of silk varieties by different techniques. J. Text. Inst..

[41] Padaki N.V., Das B., Thirumalesh R.M. (2015). Enzyme applications in silk processing. Adv. Silk Sci. Tech..

[42] Nakpathom M., Somboon B., Narumol N. (2009). Papain enzymatic degumming of Thai *Bombyx mori* silk fibers. J. Microsc..

[43] Feng Y., Li X., Li M., Ye D., Zhang Q., You R., Xu W. (2017). Facile preparation of biocompatible silk fibroin/cellulose nanocomposite films with high mechanical performance. ACS Sustain. Chem. Eng..

[44] Zhou G., Shao Z., Knight D.P., Yan J., Chen X. (2009). Silk fibers extruded artificially from aqueous solution of regenerated *bombyx mori* silk fibroin are tougher than their natural counterparts. Adv. Mater..

[45] You R., Zhang Y., Liu Y., Liu G., Li M. (2013). The degradation behavior of silk fibroin derived from different ionic liquid solvents. Nat. Sci..

[46] Zhou C.Z., Confalonieri F., Jacquet M., Perasso R., Li Z.G., Janin J. (2001). Silk fibroin: Structural implications of a remarkable amino acid sequence. Proteins.

[47] Wang X., Ding Z., Wang C., Chen X., Xu H., Lu Q., Kaplan D.L. (2018). Bioactive silk hydrogels with tunable mechanical properties. J. Mater. Chem. B.

[48] Elahi M.F., Guan G., Wang L., King M.W. (2014). Improved hemocompatibility of silk fibroin fabric using layer-by-layer polyelectrolyte deposition and heparin immobilization. J. Appl. Polym. Sci..

[49] Wang X., Qiu Y., Carr A.J., Triffitt J.T., Sabokbar A., Xia Z. (2011). Improved human tenocyte proliferation and differentiation in vitro by optimized silk degumming. Biomed. Mater..

[50] Vepari C., Kaplan D.L. (2007). Silk as a biomaterial. Prog. Polym. Sci..

[51] Lawrence B.D., Omenetto F., Chui K., Kaplan D.L. (2008). Processing methods to control silk fibroin film biomaterial features. J. Mater. Sci..

[52] Wang H.Y., Zhang Y.Q. (2013). Effect of regeneration of liquid silk fibroin on its structure and characterization. Soft Matter.

[53] Allardyce B.J., Rajkhowa R., Dilley R.J., Atlas M.D., Kaur J., Wang X. (2016). The impact of degumming conditions on the properties of silk films for biomedical applications. Text. Res. J..

[54] Yamada H., Nakao H., Takasu Y., Tsubouchi K. (2001). Preparation of undegraded native molecular fibroin solution from silkworm cocoons. Mater. Sci. Eng. C.

[55] Inoue S., Tanaka K., Arisaka F., Kimura S., Ohtomo K., Mizuno S. (2000). Silk fibroin of *Bombyx mori* is secreted, assembling a high molecular mass elementary unit consisting of H-chain, L-chain, and P25, with a 6:6:1 molar ratio. J. Biol. Chem..

[56] Wang Z., Yang H., Li W., Li C. (2018). Effect of silk degumming on the structure and properties of silk fibroin. J. Text. Inst..

[57] Gulrajani M.L. (1992). Degumming of silk. Rev. Prog. Color. Relat. Top..

[58] Jiang P., Liu H., Wang C., Wu L., Huang J., Guo C. (2006). Tensile behavior and morphology of differently degummed silkworm (*Bombyx mori*) cocoon silk fibres. Mater. Lett..

[59] Perez-Rigueiro J., Viney C., Llorca J., Elices M. (2000). Mechanical properties of silkworm silk in liquid media. Polymer.

